# Evaluation of DNA Methylation Changes and Micronuclei in Workers Exposed to a Construction Environment

**DOI:** 10.3390/ijerph16060902

**Published:** 2019-03-13

**Authors:** Isana R. Silva, Manoela C. A. S. Ramos, Lídia M. R. B. Arantes, André V. H. Lengert, Marco A. Oliveira, Fernanda P. Cury, Guilherme Martins Pereira, Aldenor G. Santos, Fernando Barbosa, Pérola C. Vasconcellos, Cyrille Cuenin, Zdenko Herceg, Henrique C.S. Silveira

**Affiliations:** 1 Molecular Oncology Research Center, Barretos Cancer Hospital, Barretos 14784-400, São Paulo, Brazil; isanateclab@hotmail.com (I.R.S.); manoelaaguena@gmail.com (M.C.A.S.R.); lirebolho@hotmail.com (L.M.R.B.A.); ahlengert@gmail.com (A.V.H.L.); fernandacury_7@hotmail.com (F.P.C.); 2 Center for Research Support (NAP), Barretos Cancer Hospital, Barretos 14784-400, São Paulo, Brazil; marco.bioestatistica@hcancerbarretos.com.br; 3 Instituto de Química—USP, São Paulo 05508-000, Brazil; martinspereira2@hotmail.com (G.M.P.); perola@iq.usp.br (P.C.V.); 4 Instituto de Química—UFBA, Salvador 40170-115, Bahia, Brazil; gomes_aldo@hotmail.com; 5 School of Pharmaceutical Sciences, University of São Paulo, Ribeirão Preto 14040-903, São Paulo, Brazil; fbarbosa@fcfrp.usp.br; 6 Epigenetics Group, International Agency for Research on Cancer (IARC), 150 Cours Albert-Thomas, 69008 Lyon, France; cueninc@iarc.fr (C.C.); HercegZ@iarc.fr (Z.H.); 7 University of Cuiabá, Campus Beira Rio, Cuiabá 78008-000, Mato Grosso, Brazil

**Keywords:** construction workers, DNA methylation, micronuclei, metals, polycyclic aromatic hydrocarbons

## Abstract

Methylation levels in tumor-suppressor genes and repetitive sequences have previously been used to study the relationship between environmental air pollution and epigenetic changes related to cancer. In this study, we measured the methylation profiles of the promoter regions *CDKN2A*, *MLH1* and *APC* and the repetitive sequence *LINE-1* in 59 workers exposed to the construction environment and in 49 unexposed workers. We also evaluated the micronuclei frequency and levels of trace elements in the blood of all workers. We evaluated of levels of particulate matter and polycyclic aromatic hydrocarbons (PAHs) at the construction site to characterize the environmental exposure. Our findings demonstrated that exposed workers exhibited significantly higher average levels of promoter methylation of *CDKN2A*, *APC*, and *MLH1* genes and increased hypomethylation of the *LINE-1* in comparison to unexposed workers (all *p* < 0.05). A higher frequency of micronuclei was observed in the exposed group (2 ± 2) compared to the unexposed group (1 ± 1) with *p* < 0.001. High levels of particulate matter (51–841 μg/m^3^) and some PAHs were found in samples from the construction environment. In summary, we provide evidence of increased DNA damage and altered *DNA* methylation of exposed workers, suggesting that genomic approaches to biomonitoring may be an effective way of estimating future cancer risk for construction workers.

## 1. Introduction

Construction workers use materials composed of various substances either classified as carcinogens or potentially carcinogenic according to the International Agency for Research on Cancer. For example, crystalline silica in the form of quartz dust is often present in the air at construction sites due to concrete mixing, cutting, drilling, blasting, demolition, and pressure cleaning. Inhalation of crystalline silica can lead to silicosis and increase the risk of lung cancer and other autoimmune diseases [1]. Construction workers are sometimes also at risk of exposure to asbestos, which is related to the development of lung and pleura cancer, asbestosis, pleural and peritoneal mesothelioma, laryngeal, ovarian, colorectal, pharynx and stomach cancer, benign pleural lesions, intestinal cancer, esophageal cancer, pancreatic cancer, kidney cancer, and mesothelioma [2,3]. Asbestos exposure can occur during insulation installation, demolition, and building renovation. However, it has been suggested that the increased risk of lung cancer in bricklayers is largely attributed to exposure to crystalline silica rather than to asbestos exposure at construction sites [1].

In addition to increasing the risk of cancer, the emissions of contaminants or pollutants into the air may also affect the general health of construction workers. For example, it was recently shown that air pollution is associated with depression in the elderly [4]. Air pollutants can be categorized as gases, persistent organic pollutants, heavy metals, and particulate matter (PM) [5]. In the United States of America, the Environmental Protection Agency (EPA) conducted a general survey of sources of particulate matter (PM) emission and concluded that the construction industry generated 13% of the total amount of PM in the air. PM from this sector is likely to consist of cement powder, gypsum, lime, industrialized mortar, wood dust due to excavation, vehicle movement or wind, asbestos, and other sources [6]. Several mechanisms such as changes in gene expression, alterations to inflammatory responses, or induced oxidative stress have been proposed to explain the link between PM and the increased risk of lung cancer [7,8]. However, at the present time, a direct relationship between PM and mechanisms associated with cancer onset are poorly understood. PM is thought to exacerbate inflammatory responses, leading to the induction of local or systemic release of reactive oxygen species, which could in turn contribute to epigenetic alterations and genomic instability [9,10]. Examination of DNA alterations and genomic instability in workers with an occupational risk is a fertile area for improved understanding of how PM increases cancer risk. 

Micronuclei (MNs) are small extranuclear bodies that have been used since the 1970s to determine whether cells have undergone some sort of DNA damage. MNs originate from chromosome fragments or lagging whole chromosomes that fail to be included in the daughter nuclei during mitosis. These acentric fragments can give rise to small nuclei or MNs, which are morphologically similar to the main nuclei, but considerably smaller [11]. Analysis of MN frequency is an important tool to assess risk in populations exposed to genotoxic agents and other compounds that are hazardous to health [12].

An alternative approach for estimating cancer risk is to use an epigenetic biomarker that can identify when genetic mechanisms involved in the initiation of carcinogenesis have been altered. Analysis of DNA methylation levels in tumor-suppressor genes in occupationally exposed populations has been used as a potential biomarker of associated cancer risk. Several studies carried out in workers who were exposed to air pollutants showed there was altered methylation of *APC*, *CDKN2A*, *TP53*, *RASSF1A*, and *MGMT* genes, and of the repetitive sequences *ALU* and *LINE-1* [13,14,15,16]. Another study in a population with prolonged exposure to arsenic found an increase in DNA methylation levels in the *p16* and *MLH1* tumor-suppressor genes [17]. A study that assessed workers exposed to vinyl chloride detected promoter hypermethylation of DNA repair genes and increased chromosomal instability, as measured by evaluating the frequency of MNs [18]. 

The methylation patterns of repetitive DNA sequences, such as *ALU* and *LINE-1* elements, may be used as a quantitative epigenetic biomarker of alterations to DNA methylation [19]. In vitro and in vivo studies have shown that hypomethylation of these elements may also occur in response to exposure to environmental pollution particles. Alterations to the cellular methylation systems are thought to be associated with inflammatory reactions that can be triggered by the inhalation of airborne pollutants. These perturbations to the DNA methylation machinery may be part of a cellular adaptive response mediating the environmental effects of pollution on human health [20]. In keeping with this idea, hypomethylation of the repetitive sequence *LINE-1* was found in workers exposed to high concentrations of polycyclic aromatic hydrocarbons (PAHs), suggesting this epigenetic biomarker may be useful in assessing occupational exposure to PAHs [13].

The majority of studies involving DNA methylation markers in occupationally exposed populations have been performed on peripheral blood leukocytes, since these are the first cells to respond to inflammatory mediators released in the lungs after environmental exposure [21,22]. Furthermore, methylation of DNA in peripheral blood has been identified as a sensitive marker for acute and short-term epigenome changes [21].

This observational study includes 59 construction workers with daily exposure to pollution in a building site and 49 age-matched controls who have no exposure to a construction environment. We use a quantitative PCR-pyrosequencing assay to measure DNA methylation levels in three tumor-suppressor genes, i.e., *CDKN2A*, *MLH1*, and *APC.* We also use this methylation assay to study the long-interspersed nucleotide element (*LINE-1*) repetitive sequence that has high representation throughout the human genome. In addition, we conduct MN analyses in lymphocytes of the study group to evaluate chromosomal instability levels and to estimate genotoxic exposure. We measured environmental Polycyclic Aromatic Hydrocarbons (PAH) levels in the construction environment to quantify the pollution levels workers are exposed to on a daily basis at a typical institutional building site.

## 2. Materials and Methods 

### 2.1. Study Group and Biological Sample Collection

The research participants in this observational study included a total of 59 construction workers from the Barretos Cancer Hospital, which comprised of the exposed group and 49 age-matched administrative workers from the same institution as the unexposed controls. The study group included men over 18 years of age, construction workers, and ex-smokers over a year after quitting. We considered passive smokers to be individuals that live with tobacco smokers. All workers who are smokers; drug users; have infectious or chronic diseases (such as autoimmune diseases); or have been exposed to other agents, such as X-rays and xylene, were excluded from the study. Each participant responded to a survey with questions related to sociodemographic characteristics, occupational routines, and general health. After completing the questionnaire, an Informed Consent Form (ICF) was presented to both groups, and the subjects were informed of the potential risks of participating in the study. After signing the consent, 15 milliliters of venous blood was collected from each participant for analysis and evaluation of MNs and for DNA extraction. This study was approved by the Barretos Cancer Hospital Ethical Committee (approval no. 586/2012).

### 2.2. MNs in Lymphocytes

For MN assays in human lymphocytes, we used the protocol established by Reference [23] and adapted by this laboratory [24,25]. Blood samples were collected from construction workers and controls. Thus, there were two cultures performed for each sample. Lymphocytes were separated by Ficoll-Paque (Invitrogen, Carlsbad, CA, USA), and cultures were grown in RPMI medium with added phytohemaglutinin (Life Technologies, Carlsbad, CA, USA) at 37 °C for 72 h. After 44 h, cytochalasin B (Sigma, St. Louis, MO, USA) was added to a final concentration of 6 μg/mL [23]. Then, after cultivation for 72 h, cells were resuspended in SurePath preservative liquid (BD TriPath Imaging, Burlington, NC, USA) to ensure a single cell layer with an excellent smear. This standard fixative solution is a mixture of ethanol, methanol and isopropyl alcohol. About 1 ml of this solution was transferred to an incubation chamber mounted on a support (BD TriPath Imaging, Burlington, NC, USA) consisting of a PrepStain Settling Chamber (BD TriPath Imaging, Burlington, NC, USA) placed on the top of a glass slide. A cell suspension from each sample was dropped onto a clean glass slide. After 1 h, the slides were stained automatically, as specified by the manufacturer. The slides were previously encoded and analyzed under an optical microscope at a magnification of 1000×. Approximately six slides were prepared and identified for each individual, and 1000 binucleated cells were counted for each slide. All slides were counted twice. 

### 2.3. DNA Isolation, Bisulfite Conversion, and Pyrosequencing

DNA from all samples was extracted using a DNeasy Blood & Tissue Kit (Qiagen, Valencia, CA, USA) according to the manufacturer’s instructions. The extracted DNA was quantified by spectrophotometry using a NanoDrop 2000c (ND-1000 Spectrophotometer v.3.0.1; Thermo Fisher Scientific, Waltham, MA, USA). For sodium bisulfite conversion, 1 μg of each DNA sample was used with a bisulfite EPITECH Kit (Qiagen) following the manufacturer’s instructions.

After treatment with sodium bisulfite conversion, each sample was subjected to polymerase chain reaction (PCR) with biotin-labeled primers. PCR was performed in a volume of 50 μL containing 2 μL converted DNA, 25 μL HotStarTaq Master Mix (Qiagen), and 1 μL of each primer (forward and reverse) at a concentration of 10 μM. The primers for *CDKN2A*, *MLH1*, *APC* and *LINE-1* with the respective genomic locations of the target CpG sites in the genome are shown in Table S1. All primers were synthesized as previously described [26] and PCR products were purified using Sepharose Beads on a PyroMark Vacuum Prep Workstation (Qiagen) according to the manufacturer’s protocol and were transferred to a pyrosequencer (PyroMark Q96 ID System; Qiagen). Pyrosequencing was performed using the DNA from the samples and control template DNAs (methylated and non-methylated DNA) were obtained from (Qiagen). The methylation results were expressed as a percentage of methylated cytosines divided by the sum of methylated and unmethylated cytosines. Non-CpG cytosine residues were used to verify the efficiency of bisulfite conversion. 

### 2.4. Analysis of Trace Elements in Blood

The determination of trace elements in blood samples was carried out with an inductively coupled plasma mass spectrometer equipped with a reaction cell (ICP-MS ELAN DRCII, PerkinElmer, SCIEX, Norwalk, CT, USA) operating with high-purity argon (99.9%, Praxaair, Pinhais, Brazil) and ammonium as the reaction gas (99.9%, Praxaair, Pinhais, Brazil). The analyses were conducted at the School of Pharmaceutical Sciences of Ribeirão Preto (University of São Paulo), Ribeirão Preto, São Paulo, following a methodology previously described [27]. The method quantification limits (10 s) used for As, Cd, Co, Cr, Cu, Mn, Pb, Se, Tl, V, and Zn were 0.04, 0.01, 0.03, 0.02, 0.8, 0.03, 0.01, 0.8, 0.1, 0.02, and 2.4 µg L^−1^, respectively.

### 2.5. Analysis of PM and Work Environment

PM_2.5_ emitted in the construction environment was collected using a large-volume sampler (Hi-Vol; Energy) with quartz-fiber filters. The filters were weighed prior to and after a sampling period of 8 h, and the mass difference was used to determine the particle concentration. The particulate material was extracted from the filters by ultrasonication. Concentrations of PAHs and nitro-PAHs were determined by gas chromatography/mass spectrometry (GC/MS; Varian 3800 HD) as described [28]. To determine the compounds present, an analysis was performed in the pooled material present in all the filters.

The exposed group was drawn from the construction team at Barretos Cancer Hospital. They worked in a well-ventilated renovation site occupying one floor of the hospital comprising 1895 m^2^. This work environment involved all stages of the construction process, including demolition, building, painting, and welding. In addition to substances related to the construction process, workers were exposed to periodic diesel fuel emissions from vehicles delivering to the site. Face masks were provided for use during demolition and when the construction activities generated airborne dust and fine cement particles. The controls were drawn from administrative workers from the Barretos Cancer Hospital who worked in an air-conditioned office environment with no exposure to the construction site substances.

### 2.6. Statistical Analysis

SPSS software version 21.0 for Windows was used for statistical analyses. Initially, the data were tabulated considering the descriptive statistics (mean, standard deviation, minimum, maximum, and quartiles) for quantitative data and frequency tables for qualitative data. To analyze the frequencies of MNs in lymphocytes for the two groups, nonparametric Mann-Whitney tests were applied. Regression Poisson analyses were performed to analyze MN frequencies. 

For the analysis of DNA methylation of exposed and unexposed groups, parametric or nonparametric distributions were evaluated in both groups and were used for *t*-tests and Mann-Whitney statistical tests, respectively. For DNA methylation analyses, means and/or medians of each gene for each group were calculated, and the differences were also determined for each site for each gene. After analysis, the *p values* were adjusted by the Benjamini and Hochberg method and multiple comparison corrections were performed with R environment considering 21 CpG sites interrogated in this study. For correlation analyses between DNA methylation profiles of genes that were significant, the group of construction workers was divided by the mean, according to drinking habits and passive smoking information. From this division, we used *t*-tests and/or the Mann-Whitney tests for statistical evaluation. For correlations analyses between DNA methylation profiles of genes that were significant and exposition to occupational agents in the actual work environment, only the methylation levels from construction workers were considered and we used *t*-tests and/or the Mann-Whitney testes for statistical evaluation. To evaluate the influence of changes in the methylation level in the exposed group with regard to previous smoking habits, exposure to passive smoking, age, and alcohol consumption, we conducted multiple linear regression analyses considering these variables. 

For the analysis of trace elements determined by ICP-MS, a nonparametric test was used (Mann–Whitney) and was conducted to examine differences in the mean levels in blood for Cu, Zn, Cr, Vu, As, Pb, Cd, Se, Mn, Co and Ti in the construction workers and the control group. Values lower than the LOQ (limit of quantification) were set as LOQ/2 for the statistical analysis.

## 3. Results

### 3.1. General Characteristics

The average age of the study participants was 39 years for the workers in the exposed construction group and 32 years for the unexposed control administrative group (ranges of 13 and 10 years, respectively). All participants (100%) were nonsmokers. Most participants (61% of exposed cases and 83% of controls) consumed alcohol. In addition, 62.7% of exposed cases and 31.1% of controls were classified as passive smokers (Table 1). To establish what types of materials and other environmental substances were usually present at the site, we questioned the construction workers by interview. The following substances were reported: sand (70.7% were exposed), concrete (83.1%), silica (33.9%), wood dust (41.4%), and ultraviolet radiation (84.7%).

### 3.2. Analysis of Trace Elements in Blood

After, levels of trace elements in blood from the construction workers and the control group were determined. The nonparametric test showed significant differences in the means of major of analytes examined; only titanium trace levels did not show statistical differences between groups (Table S2). The means were higher in the control group in comparison with construction workers.

### 3.3. Analysis of PM_2.5_ Emitted in the Construction Environment

The fine particulate matter (particles lower or equal to 2.5 micrometers) concentrations of the samples ranged from 51 to 841 μg/m^3^. The average concentrations of polycyclic aromatic hydrocarbons (PAHs) in the PM_2.5_ ranged from 0.01 (dibenzanthracene [DBA]) to 0.6 ng/m^3^ (acenaphthene), as shown in Table S3. The most abundant PAHs were acenaphthene (18%), phenanthrene (17%), pyrene (12%), and fluoranthene (10%). Benzo(a)pyrene, a highly carcinogenic and the most extensively studied PAH, represented 3% of all PAHs.

The benzo(a)pyrene-equivalent index was calculated by the following equation: BaPE = BaA × 0.06 + B(b + k)F × 0.07 + BaP + DBA × 0.6 + InP × 0.08 [29] and was on average equal to 0.18 ng/m^3^ in our samples. This value was lower than that considered to indicate a risk of cancer (1 ng/m^3^) [29]. Species considered carcinogenic (Benzo(a)anthracene, benzo(b)fluorathene, benzo(k)fluorathene, benzo(a)pyrene, dibenzo(a,h)anthracene and indeno(c,d)pyrene) ranged from 12% to 28% of the total PAHs.

Nine nitro-PAHs were found in the samples, including 1-nitropyrene, a highly mutagenic compound which arises from the combustion of diesel fuel. The total concentration of nitro-PAH was on average 2.5 ng/m^3^, and the most abundant PAHs were 6-nitrobenzo(a)pyrene (1.0 ng/m^3^), 7-nitrobenzo(a)anthracene (0.55 ng/m^3^), and 1-nitropyrene (0.55 ng/m^3^), as demonstrated in Table S3. In some cases, emission sources can be inferred. The fluoranthene/pyrene ratio was on average 0.79, and BbF + BKF/BPE was equal to 0.95, suggesting the influence of vehicle exhaust at the construction site [30,31].

### 3.4. Tumor-Suppressor Genes and LINE-1 Methylation Levels by Exposure Group

Next, we compared the DNA methylation levels in different CpG sites of the promoter regions of *CDKN2A*, *MLH1*, and *APC* genes and in the genome-wide repetitive sequence *LINE-1* in samples from the exposed and unexposed groups (Table 2). Of the seven sites analyzed in the promoter region of *CDKN2A*, only sites 2 and 7 showed statistically significant differences between the groups (*p* < 0.05). When the seven CpG sites were analyzed together, we observed that the average methylation level of the *CDKN2A* promoter region was significantly higher in the exposed group than in the unexposed control group (4.79% versus 3.63%, *p* < 0.05). For analysis of the promoter region of the *MLH1* gene, six CpG sites were evaluated; among these sites, sites 3–6 showed significantly increased average methylation levels (*p* < 0.02). Moreover, the overall average of all CpGs was significantly different between the exposed and unexposed groups (4.5% versus 5.04%, *p* < 0.007). For the methylation levels of the promoter region of the *APC* gene, a total of three CpG sites were evaluated; however, we did not observe a statistically significant difference between the exposed and unexposed groups (all CpG sites combined). Nevertheless, in the analyses of methylation levels at the individual CpG sites, we found two sites that showed significant differences between exposed and unexposed groups, namely, site 2 (*p* < 0.002) and site 3 (*p* < 0.013). This may indicate that methylation at these CpG sites was involved in chromatin changes related to the regulation of gene expression. Therefore, the calculations were performed again for the CpG sites that showed significant differences. We observed an increased mean (4.29%) and median (4.29%, range: 0.81–9.61) for the construction workers group in comparison to the control group (mean: 3.11%, median: 2.61 [range: 0–10.82]; *p* < 0.001). In the analysis of the five CpG sites of *LINE-1* sequence, two CpG locations (site 1, *p* = 0.01; site 5, *p* = 0.005) showed significant differences between the exposed and unexposed groups. Moreover, considering all sites, a significant difference (*p* < 0.05) was observed (Table 2).

### 3.5. Substances from the Work Environment and Their Effects on DNA Methylation 

The questionnaire provided information on the most frequent agents and substances to which construction workers were exposed in the workplace. Therefore, stratification analyses were performed to evaluate the possible influence of exposure to agents found in the workplace on the gene methylation profile. The only significant findings were exposure to wood dust (*p* < 0.02) and silica (*p* < 0.05), which were found to influence the average methylation levels of the *MLH1* gene and *LINE-1* repeat sequence, respectively (Table S4).

### 3.6. Confounding Factors and DNA Methylation Levels

In order to assess changes in methylation levels in the exposed group with regard to previous smoking habits, exposure to passive smoking, and alcohol consumption, we conducted multiple linear regression analyses considering these variables. Thus, we fit a model for each analyzed gene using the average percentage of DNA methylation as the response. For the *MLH1* and *APC* genes and the *LINE-1* repeat sequence, we observed that only the variable “group” showed statistically significant values when analyzed in conjunction with other variables. When analyzed in conjunction with the four variables, the CDKN2A gene approached significance (*p* = 0.06). The other variables (ex-smoker, passive smoker, and drinking habit) had higher values (*p* =0.18, *p* = 0.70, and *p* = 0.22), respectively, Table 3.

Based on the multiple linear regression model, we found that the methylation levels of all genes were significantly influenced by the “group” (exposure), while all other variables remained constant. When we analyzed the independent groups, keeping the variables constant, we found that *CDKN2A*, *MLH1*, and *APC* genes showed higher mean percentages of methylation in the exposed group, suggesting once again that the methylation levels of these genes were influenced by occupational exposure rather than other confounding variables. The same findings were observed for the *LINE-1* repeat sequence. When the variables were kept constant and only the group was analyzed, methylation levels in the exposed group were lower, again suggesting that exposure influenced the methylation level.

### 3.7. MN Frequencies and Correlations with DNA Methylation

**Figure 1:**
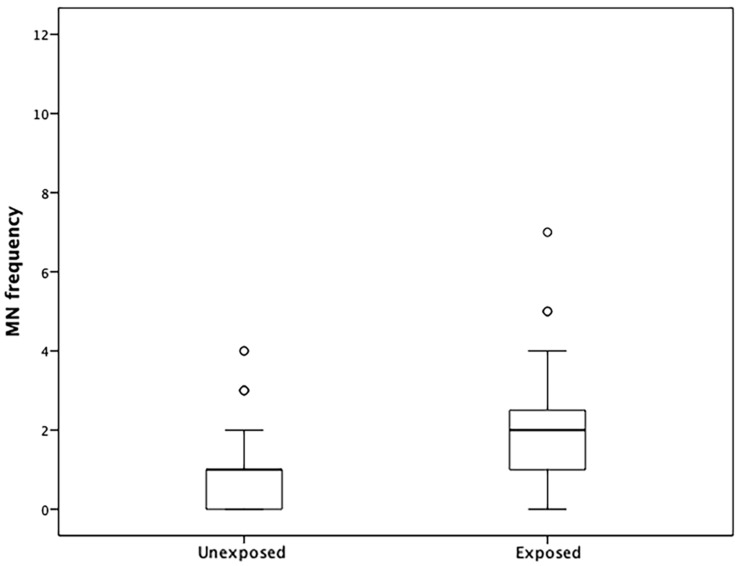
Graphic representation of MN frequency distributions in unexposed and exposed groups. Circles indicate outliers, bars indicate percentile values, and lines indicate minimum and maximum standard deviations.

We observed a higher frequency of MNs in lymphocytes in the exposed group than in the unexposed group (Figure 1). Poisson regression analysis revealed a relationship between MN frequency and methylation levels in the *LINE-1* repeat sequence of the exposed group (Table 4). No correlation was established for the *APC*, *MLH1*, and *CDKN2A* genes.

## 4. Discussion

In the present study, we assessed the DNA methylation profiles of the promoter regions of *APC*, *CDKN2A*, and *MLH1* genes and CpG sites from the *LINE-1* repeat sequence in peripheral blood leukocytes of construction workers using pyrosequencing. This molecular method has the advantage of producing individual measurements in more than one CpG dinucleotide, providing a more precise indication of DNA methylation in the studied genomic regions. This method was chosen as it is both quantitative and qualitative and is well-controlled for the effectiveness of bisulfite conversion. Also, we used a cellular assay of genotoxicity, the MN frequency, which allowed us to evaluate MN frequencies in construction workers and controls and compare findings to the trace elements in blood and the characterization of PM_2.5_ in the work environment.

Previous studies of methylation levels on the same genes we studied have shown them to be altered in the blood of patients with lung cancer and leukocytes collected from blood-exposed populations to particulate matter [15,18]. A recent study of individuals exposed to arsenic in their water supply found increased DNA methylation levels of *MLH1* and *CDKN2A* genes and decreased the methylation level of the repetitive sequence *LINE-1* [17]. In this study, the authors used multiple linear regression analyses to show there was a positive association between the arsenic fraction in the urine and levels of *CDKN2A* (*p16)* methylation. The *CDKN2A* gene is a cell cycle regulator, and hypermethylation of this gene has been found in colon, liver, skin, and lung tumors [17,32]. Additionally, hypermethylation of *CDNK2A* has been found not only in tumor tissue but also in leukocytes of individuals exposed to arsenic, chromate, tobacco, radon, particulate material containing PAHs, and radiation [15,16,17,32]. Our results corroborate these studies, as hypermethylation of the *CDKN2A* gene was significantly different in peripheral blood leukocytes of the construction workers in comparison to the control group. 

*MLH1*, a member of the mismatch repair (*MMR*) gene family, is involved in maintaining genomic stability [17]. The methylation levels of the *MLH1* gene were found to be significantly higher in individuals exposed to arsenic in their water supply [17]. In another study of *MLH1* chromosomal instability, as evaluated by MNs, methylation of the *MLH1* promoter region in workers exposed to vinyl chloride (plastic) was analyzed [18]. The authors found that methylation of the *MLH1* gene was not significantly altered in exposed individuals, but there was increased chromosomal damage as determined by MNs. Our results based on construction site pollution showed significant methylation of the *MLH1* gene in exposed versus unexposed control participants.

In this study, the *APC* gene exhibited higher methylation in exposed workers than in controls. *APC* hypermethylation occurs during the early stages of tumorigenesis and has been extensively studied in lung cancer [15]. Increased methylation levels have been detected in *APC*, *MLH1*, and *CDKN2A* genes in industrial workers with lung cancer [33]. In this study, increased methylation was found in the lung tumors from patients with a confirmed history of exposure to chromium. The lung tumors from patients without the same occupational history did not have increased methylation, suggesting that lung cancer caused by exposure to chromium may be connected to progressive methylation of some of the studied tumor suppressor genes. A previous study [34] found increased methylation of *CDKN2A* and *APC* and decreased methylation of the *LINE-1* repetitive sequence in the lungs of rats exposed to PM from vehicle smoke. Our results also showed increased DNA methylation in these tumor suppressor genes and a significant decrease in the methylation of *LINE-1* in construction workers exposed to a mixture of agents in the workplace. These data suggested that the set of agents in the work environment could be associated with changes in DNA methylation levels. Moreover, this mixture may be related to genomic instability, as previously described by others [25].

*LINE-1* repetitive sequences are retrotransposons highly repeated throughout the human genome. Several studies have found that altered methylation of *LINE-1* is associated with cancer susceptibility and poor outcomes [35,36]. Hypomethylation of *LINE-1* sequences in leukocytes is associated with an increased risk of various types of cancer [37]. Salas and colleagues [38] evaluated individuals exposed to the solvent trihalomethane, a class of disinfection byproducts, and found a positive correlation between the methylation levels of *LINE-1* and exposure to this solvent. Our Poisson regression analyses indicated that exposed workers with lower LINE-1 methylation may have a higher number of MNs (IRR = 0.89 [0.8–0.99]; *p* = 0.039) than controls. This observation suggests that changes in methylation levels of *LINE-1* are also associated with genomic instability. 

MN frequency in lymphocytes was higher in workers exposed to the construction environment than in controls. Previous results from our group have shown differences in MN frequencies in the buccal mucosa cells of construction workers in comparison to controls [25]. Additionally, MN frequencies have been measured in several classes of workers, and increased MN frequency in exposed populations is considered a risk factor for cancer [12,39]. Hoyos-Giraldo and colleagues [40] measured MN frequency and methylation profiles of the promoter regions of *GSTP1*, *CDKN2A*, *APC*, and *CDH1* genes in exfoliated urothelial cells of workers involved in painting cars and showed that the group exposed to paint solvents had higher frequencies of MN and higher percentages of methylation in *GSTP1*, *CDKN2A*, and *APC* genes. Moreover, Poisson regression analyses indicated that exposed workers with methylated regions in *GSTP1* and *CDKN2A* also had increased MN frequencies in comparison to controls.

Recent studies have demonstrated that exposure to metals could be associated with changes in epigenetic status [41,42]. Surprisingly, our findings concerning the levels of trace elements in blood from the construction workers and control group using ICP-MS analysis revealed that the means were higher in the control than in the construction workers group. We ascribe these differences between groups to variations in trace metals in the diet, which is known to be higher in salads, vegetables, fish, and fruits. It is possible that dietary difference between the construction workers and administrative staff may underlie the observed trace metal differences. Furthermore, the combination of higher exposure to ambient air pollutants and environmental contaminants, together with poor diet may have contributed to oxidative stress and activation of inflammatory pathways, which increases susceptibility to DNA methylation changes [43].

In order to characterize the environment of which the workers were exposed to, we characterized PM_2.5_. The concentrations of fine PM in the samples ranged from 51 to 841 µg/m^3^, which was much higher than recommended by the World Health Organization (25 µg/m^3^, daily average). Furthermore, nine nitro-PAHs were evaluated, and the total concentration of nitro-PAHs was on average 2.5 ng/m^3^. The concentrations of PM_2.5_ found in another study that compared urban and biomass burning sites from samples collected in other Brazilian cities, such as Araraquara and Piracicaba (also located in Sao Paulo State) were lower than levels that we report in this study from a construction environment at this Hospital [44].

## 5. Conclusions

In summary, our results demonstrate that variation in DNA methylation levels in tumor-suppressor genes such as *CDKN2A*, *APC*, and *MLH1*, as well as in the *LINE-1* repeat sequence in peripheral blood leukocytes of construction workers could be a valuable indicator of specific exposure and adverse health effects. Altered DNA methylation could be used as a potential biomarker for the early detection of changes in occupationally exposed individuals. One important limitation of our study is that we did not have an opportunity to evaluate the influence of diet and nutritional intake on the participants. Since it is known that diet can alter the methylation profile and our analysis demonstrated that trace elements were higher in control group, it is possible that dietary factors may also be involved in group comparisons. Nevertheless, we found higher MN frequencies in the construction worker group, demonstrating the genotoxic potential of this work environment. We also observed a high PM index (higher than the allowed by the WHO) in the construction environment. During our analysis, we detected the presence of low concentrations of PAHs and nitro-PAHs, which could be present in the environment through external influences. Future, more extensive studies should be carried out to validate our findings. 

## Figures and Tables

**Table 1:** Socio-demographic characteristic of participants from collected group (Exposed × Unexposed).

Variable		Exposed (N = 59)	Unexposed (N = 49)	*p* Value
Age	<35	25 (46.3)	24 (49)	0.60 ^a^
	≥35	29 (53.7)	25 (51)	
Exposure time	Average (years)	10	-	-
Passive smoker	Yes	37 (62.7)	14 (31.1)	0.001 ^a,^*
	No	22 (37.3)	31 (68.9)	
Ex- smoker	Yes	25 (47.2)	7 (22.6)	0.036 ^a,^*
	No	28 (52.8)	24(77.4)	
Alcohol consumption	Yes	36 (61)	40 (83.3)	0.01 ^a,^*
	No/Past	23 (39)	8 (16.7)	
Ethnic group	White	36 (63.2)	28 (73.7)	0.28 ^b^
	Black	8 (14)	2 (5.3)	
	Mixed	13 (22.8)	7 (18.4)	
	Asian	0	1 (2.6)	

N—Number of participants. * Statistically significant. ^a^ Chi-squared tests were used for statistical analysis. ^b^ Fisher’s exact tests were used for statistical analysis.

**Table 2:** Evaluation of the DNA methylation profiles in the promoter regions of the *CDKN2A*, *MLH1*, and *APC* genes and repetitive sequence *LINE-1* in construction workers and controls.

Cp Genomic Sites	Unexposed Workers			Exposed Workers			*p* Value	FDR ^c^ Corrected *p* Values
	N	Mean ± SD	Median (IQR)	N	Mean ± SD	Median (IQR)		
*CDKN2A* (%5mC)								
CpG site 1	44	2.85 ± 1.89	2.81 (0–5.75)	57	3.48 ± 2.57	2.97 (0−10.06)	0.48 ^b^	0.53
CpG site 2	44	5.11 ± 2.81	5.39 (0–10.35)	57	6.56 ± 3.03	6.82 (0−13.58)	0.02 ^b,^*	0.04 *
CpG site 3	44	2.51 ± 2.31	2.50 (0–6.53)	57	3.25 ± 2.57	2.81 (0−9.97)	0.24 ^b^	0.31
CpG site 4	44	4.42 ± 1.51	4.4 (0–7.58)	57	5.02 ± 2.00	5.08 (0−10.01)	0.11 ^b^	0.18
CpG site 5	44	4.12 ± 2.76	4.66 (0–9.47)	57	5.33 ± 2.86	5.3 (0−11.62)	0.08 ^b^	0.14
CpG site 6	44	1.93 ± 1.84	1.97 (0−5.14)	57	2.47 ± 2.29	2.15 (0−8.00)	0.40 ^b^	0.47
CpG site 7	44	4.46 ± 2.35	4.67 (0−8.87)	57	5.87 ± 2.37	5.68 (0−12.22)	0.005 ^b,^*	0.02 *
All CpG sites combined	44	3.63 ± 2.04	3.68 (0−7.47)	57	4.79 ± 2.57	4.44 (0−10.73)	0.05 ^b,^*	
*MLH1* (%5mC)								
CpG site 1	41	4.76 ± 1.54	4.35 (2.32−8.63)	56	5.20 ± 1.48	5.26 (0−8.10)	0.15 ^a^	0.22
CpG site 2	41	3.69 ± 1.29	3.62 (0−5.85)	56	3.70 ± 1.29	3.64 (0−6.42)	0.76 ^b^	0.76
CpG site 3	41	3.02 ± 1.80	3.27 (0−6.25)	56	3.90 ± 1.27	4.03 (0−7.25)	0.02 ^b,^*	0.04 *
CpG site 4	41	5.29 ± 2.02	5.13 (0−9.50)	56	6.18 ± 1.57	6.26 (2.53−9.70)	0.01 ^a,^*	0.03 *
CpG site 5	41	4.61 ± 1.87	4.10 (0−8.34)	56	5.90 ± 1.50	5.92 (2.42−9.59)	0.001 ^b,^*	0.01 *
CpG site 6	41	1.91 ± 2.01	2.06 (0−5.72)	56	3.86 ± 1.47	3.95 (0−8.47)	0.001 ^b,^*	0.01 *
All CpG sites combined	41	4.22 ± 1.55	4.00 (1.74−7.49)	56	5.04 ± 1.38	5.03 (1.61−9.76)	0.007 ^a,^*	
*APC* (%5mC)								
CpG site 1	47	8.78 ± 10.14	7.48 (0−68.05)	58	8.08 ± 3.26	8.13 (2.59−21.49)	0.65 ^b^	0.68
CpG site 2	47	1.99 ± 2.72	0.00 (0−9.46)	58	3.43 ± 2.21	3.57 (0−11.01)	0.002 ^b,^*	0.01 *
CpG site 3	47	4.22 ± 2.20	4.00 (0−12.18)	58	5.13 ± 1.66	5.03 (1.61−11.51)	0.013 ^b,^*	0.03 *
All CpG sites combined	47	5.47 ± 2.43	6.13 (1.29−11.54)	58	6.43 ± 3.33	5.73 (1.17−15.48)	0.29 ^b^	
CpG Hot	47	3.11 ± 2.10	2.61 (0−10.82)	58	4.29 ± 1.83	4.29 (0.81−9.61)	<0.001 ^b,^*	
*LINE-1* (%5mC)								
CpG site 1	44	74.45 ± 1.36	74.72 (70.72−76.94)	57	73.20 ± 5.08	73.83 (36.54−73.17)	0.01 ^b,^*	0.03 *
CpG site 2	44	73.42 ± 7.30	68.87 (66.92−84.14)	57	72.52 ± 7.78	67.84 (64.95−85.26)	0.16 ^b^	0.22
CpG site 3	44	63.13 ± 6.68	58.82 (55.87−73.13)	57	62.00 ± 7.16	58.20 (49.44−74.35)	0.08 ^b^	0.14
CpG site 4	44	59.08 ± 5.04	62.30 (50.52−64.39)	57	59.17 ± 4.38	61.24 (48.66−64.35)	0.34 ^b^	0.42
CpG site 5	44	66.18 ± 2.41	66.47 (57.53−72.28)	57	67.26 ± 1.11	67.14 (63.51−70.59)	0.005 ^b,^*	0.02 *
All CpG sites combined	44	68.04 ± 1.55	68.63 (64.98−70.37)	57	66.83 ± 2.33	65.93 (62.48−72.48)	0.005 ^b,^*	

N varied based on the success of pyrosequencing assay. ^a^ *t*-tests. ^b^ Mann-Whitney tests. ^c^ False Discovery Rate. * Statistically significant.

**Table 3:** Multiple linear regression model for the methylation percentages (%) of the *CDKN2A*, *MLH1*, and *APC* genes and the *LINE-1* repeat sequence in relation to passive smoking, drinking habit, and ex-smoker status (group case × control).

Gene	Variable	Β	SD (β)	*p* Value
*CDKN2A* (n = 101)	Constant	2.69	0.74	<0.001 *
	Group (case/control)	1.14	0.61	0.06
	Ex-smoker (yes/no)	0.82	0.61	0.18
	Passive smoker (yes/no)	0.23	0.6	0.7
	Drinking habit	0.72	0.59	0.22
*MLH1* (n = 97)	Constant	2	0.55	<0.001 *
	Group (case/control)	1.86	0.45	<0.001 *
	Ex-smoker (yes/no)	0.13	0.45	0.78
	Passive smoker (yes/no)	−0.07	0.44	0.88
	Drinking habit	0.01	0.43	0.98
*APC* (n = 105)	Constant	3.33	0.61	<0.001 *
	Group (case/control)	1.05	0.5	0.03 *
	Ex-smoker (yes/no)	0.44	0.51	0.39
	Passive smoker (yes/no)	−0.44	0.49	0.37
	Drinking habit	0.16	0.49	0.74
*LINE-1* (n = 101)	Constant	67.22	0.64	<0.001 *
	Group (case/control)	−1.12	0.53	0.03 *
	Ex-smoker (yes/no)	−0.17	0.53	0.75
	Passive smoker (yes/no)	0.46	0.52	0.38
	Drinking habit	0.69	0.51	0.18

* Statistically significant.

**Table 4:** Correlations among repetitive sequence *LINE-1* methylation, MN frequencies, and construction environment by Poisson regression analysis.

Groups	Gene	N	B	IRR (CI95%)	*p* Value
Exposed	LINE-1	48	−0.114	0.89 (0.8–0.99)	0.039
Unexposed	LINE-1	40	0.142	1.15 (0.93–1.43)	0.196

## Supplementary Materials

The following are available online at https://www.mdpi.com/1660-4601/16/6/902/s1, Table S1: Primer sequences and gene regions analyzed, Table S2: Levels of trace elements in blood of the construction workers in comparison to the control group, Table S3: Characterization of PAHs found in PM (PM_2.5_) at the construction site, Table S4: Methylation level of the *MLH1* gene and the repetitive sequence *LINE-1*, analyzed with regard to exposure to occupational agents in the actual work environment.
