# Supplementary material for: Evaluation of DNA Methylation Changes and Micronuclei in Workers Exposed to a Construction Environment
Source: Int J Environ Res Public Health. 2019 Mar 13;16(6):902. doi: 10.3390/ijerph16060902 (PMC6466300; doi:10.3390/ijerph16060902)
Supplement: Supplementary file 1 [file ijerph-16-00902-s001.pdf]

Table S1. Primer sequences and gene regions analyzed

Gene	Primers	T (°C)	Amplicon (bp)	Analyzed sequence
APC	Forward - TTTTGTTTGTTGGGGATTG<br>Reverse Biot.- CTCCAACACCTACCCCATTT<br>Sequencing – GGGGTTTTGTGTTTTA<br>Chromosome Loc.: chr5:112073325-112073635	55	311	TTGC/TGGAGTGC/TGGGTC<br>/TGGGAAGC/TGGAG
MLH1	Forward - TTTAGGAGTGAAGGAGGT<br>Reverse Biot. - CCCTATACCTAATCTATC<br>Sequencing–GTTTTGAGTAGAGTTTTATTAGGGT<br>Chromosome Loc: chr3:37034287-37034459	51	173	C/TGC/TGC/TGTTC/TGTC/T<br>GTTC/TGTTATATATC/TGTT
LINE-1	Forward Biot - TAGGGAGTGTTAGATAGTGG<br>Reverse - AACTCCCTAACCCCTTAC<br>Sequencing – CAAATAAAACAATACCTC	58	120	G/ACCCTACTTCG/AACTC<br>G/ACG/ACACG/AATAC
CDKN2A	Forward - GAGGGGTTGGTTGGTTATTAGA<br>Reverse Biot - TACAAACCCTCTACCCACCTAAAT<br>Sequencing – TGGTTATTAGAGGGTG<br>Chromosome Loc.: chr9:21974659-21974920	65	262	GGGC/TGGATC/TGC/TGTG<br>C/TGTTC/TGGC/TGGTTGC/<br>TGGAGA

T: temperature

Table S2. Levels of trace elements in blood of the construction workers in comparison to the control group

	Control (N=47)			Case (N=56)			
**Analytes**	**Mean (±SD)**	**Min**	**Max**	**Mean (±SD)**	**Min**	**Max**	**p-value**
Cu µg/L	1516 (247)	1019	2301	1274 (285)	778	2684	0.001
Zn µg/L	10514 (2501)	6145	16306	7884 (2050)	5093	14013	0.001
Cr µg/L	0.50 (1.35)	0.01	8.72	0.14 (0.37)	0.01	2.21	0.001
Vu µg/L	0.26 (0.21)	0.01	0.85	0.14 (0.09)	0.01	0.57	0.002
As µg/L	10.40 (10.93)	2.71	49.34	3.57 (1.15)	2.05	7.97	0.001
Pb µg/L	0.17 (0.57)	0.01	3.80	0.04 (0.12)	0.01	0.7	0.002
Cd µg/L	0.17 (0.57)	0.01	3.80	0.04 (0.12)	0.01	0.7	0.002
Se µg/L	89.49 (26.80)	60	173	64.13 (18.70)	45.11	142	0.001
Mn µg/L	3.02 (3)	0.01	10.70	0.81 (2.11)	0.01	12.40	0.001
Co µg/L	0.19 (0.32)	0.00	1.30	0.07 (0.22)	0.00	1.57	0.001
Ti µg/L	0.10 (0.14)	0.00	0.61	0.06 (0.12)	0.00	0.92	0.661

Mann-Whitney test was used for statistical analyses

Table S3. Characterization of PAHs found in PM (PM2.5) at the construction site

Polycyclic aromatic hydrocarbon (PAHs)	Sampling periods		
	Sampling I	Sampling II	Sampling III
		Concentration	
Acenaphthylene	0.05	0.08	0.03
Acenaphthene	0.23	0.62	0.03
Fluorene	0.08	0.24	0.03
Phenanthrene	0.25	0.51	0.1
Anthracene	0.06	0.18	0.03
Fluoranthene	0.13	0.28	0.07
Pyrene	0.19	0.33	0.1
Benzo[a]anthracene	0.04	0.11	0.04
Chrysene	0.06	0.12	0.04
Benzo[b]fluoranthene	0.05	0.08	0.05
Benzo[k]fluoranthene	0.02	0.04	0.03
Benzo[a]pyrene	0.05	0.06	0.04
Indeno[1,2,3-cd]pyrene	0.05	0.03	0.03
DBA	0.01	0.01	0.01
Benzoperylene	0.13	0.11	0.06
Coronene	0.03	0.01	0.02
Nitro-PAH			
1-Nitronaphthalene	0.23	-	-
1-Methyl-4-nitronaphthalene	-	-	-
2-Nitronaphthalene	0.11	-	-

2-Nitrobiphenyl	-	-	-
1-Methyl-5-nitronaphthalene	-	0.16	-
1-Methyl-6-nitronaphthalene	-	0.17	-
2-Methyl-4-nitronaphthalene	-	-	-
3-Nitrobiphenyl	-	-	-
4-Nitrobiphenyl	-	-	-
5-Nitroacenaphthene	-	-	-
2-Nitrofluorene	-	-	0.38
2-Nitrophenanthrene	-	-	0.14
3-Nitrophenanthrene	-	-	-
9-Nitrophenanthrene	-	-	-
2-Nitroanthracene	-	-	-
9-Nitroanthracene	-	-	-
2-Nitrofluoranthene	-	-	-
3-Nitrofluoranthene	-	-	-
1-Nitropyrene	0.87	0.38	0.25
2-Nitropyrene	-	-	-
4-Nitropyrene	-	-	-
7-Nitrobenzo[a]anthracene	0.55	0.64	0.47
6-Nitrocrisene	-	-	-
3-Nitrobenzanthrone	-	-	-
6-Nitrobenzo[a]pyrene	1.07	1.27	0.65
1-Nitrobenzo[e]pyrene	-	-	-

3-Nitrobenzo[e]pyrene	-	-	-
Benzoquinone	-	-	-
1,4 -Naphthoquinone	-	-	-
1,2 -Naphthoquinone	-	-	-
9,10 - Anthraquinone	-	0.12	0.05
9,10 -Phenanthraquinone	-	-	-

Table S4. Methylation level of the *MLH1* gene and the repetitive sequence *LINE-1*, analyzed with regard to exposure to occupational agents in the actual work environment.

Gene/occupational agent		Exposed workers			
		Nª	Mean ± SD	Median (IQR)	*p* value
MLH1					
Sand	Yes	15	3.62 ± 1.40	3.71 (0 - 5.55)	0.49
	No	40	3.96 ± 1.52	4.13 (0 - 8.47)	
Concrete	Yes	10	3.87 ± 0.89	3.90 (2.70 - 4.94)	0.80
	No	46	3.86 ± 1.58	3.95 (0 - 8.47)	
Wood	Yes	32	3.54 ± 1.70	3.38 (0 - 8.47)	0.02*
	No	23	4.35 ± 0.97	4.43 (2.22 - 6.16)	
Silica	Yes	36	3.85 ± 1.65	4.03 (0 - 8.47)	0.86
	No	20	3.90 ± 1.14	3.66 (2.22 - 6.16)	
Ultraviolet	Yes	7	3.31 ± 1.72	3.36 (0 - 5.28)	0.42
	No	49	3.94 ± 1.44	4.01 (0 - 8.47)	
LINE-1					
Sand	Yes	16	67.27(2.01)	66.51 (65.03 - 70.53)	0.15
	No	40	66.68(2.47)	65.68 (62.48 - 72.48)	
Concrete	Yes	10	67.22(2.30)	66.36 (65.15 - 70.61)	0.38

	No	47	66.75(2.35)	65.86 (62.48 - 72.48)	
Wood	Yes	33	67.34(2.60)	66.43 (62.48 - 72.48)	0.15
	No	23	66.19(1.75)	65.72 (63.70 - 70.62)	
Silica	Yes	37	67.21(2.35)	66.49 (62.48 - 72.48)	0.05*
	No	20	66.14(2.19)	65.54 (62.79 - 70.49)	
Ultraviolet	Yes	8	66.68(2.07)	65.56 (65.03 - 70.26)	0.81
	No	49	66.86(2.39)	66.00 (62.48 – 72.48)	

* Statistically significant

[a] Mann-Whitney tests
